# Assessment of a Single Decoupling Alchemical Approach for the Calculation of the Absolute Binding Free Energies of Protein-Peptide Complexes

**DOI:** 10.3389/fmolb.2018.00022

**Published:** 2018-03-08

**Authors:** Denise Kilburg, Emilio Gallicchio

**Affiliations:** ^1^Department of Chemistry, Brooklyn College, Brooklyn, NY, United States; ^2^Ph.D. Program in Chemistry, The Graduate Center, City University of New York, New York, NY, United States; ^3^Ph.D. Program in Biochemistry, The Graduate Center, City University of New York, New York, NY, United States

**Keywords:** protein-peptide complexes, binding free energy, alchemical calculations, single-decoupling, implicit solvent, LEDGF/p75, HIV integrase

## Abstract

The computational modeling of peptide inhibitors to target protein-protein binding interfaces is growing in interest as these are often too large, too shallow, and too feature-less for conventional small molecule compounds. Here, we present a rare successful application of an alchemical binding free energy method for the calculation of converged absolute binding free energies of a series of protein-peptide complexes. Specifically, we report the binding free energies of a series of cyclic peptides derived from the LEDGF/p75 protein to the integrase receptor of the HIV1 virus. The simulations recapitulate the effect of mutations relative to the wild-type binding motif of LEDGF/p75, providing structural, energetic and dynamical interpretations of the observed trends. The equilibration and convergence of the calculations are carefully analyzed. Convergence is aided by the adoption of a single-decoupling alchemical approach with implicit solvation, which circumvents the convergence difficulties of conventional double-decoupling protocols. We hereby present the single-decoupling methodology and critically evaluate its advantages and limitations. We also discuss some of the challenges and potential pitfalls of binding free energy calculations for complex molecular systems which have generally limited their applicability to the quantitative study of protein-peptide binding equilibria.

## 1. Introduction

Protein-protein interactions are pervasive in biological systems as they drive and regulate critical functions within the cell (Kastritis and Bonvin, 2013). Concomitantly, there is a strong interest in developing therapeutic drugs targeting protein-protein interactions (Higueruelo et al., 2009; Basse et al., 2013; Labb et al., 2013; Arkin et al., 2014). The rationale of using small-molecules or peptides to influence protein-protein binding rests on the observation that, even though they may involve large and complex molecular assemblies consisting of thousands of atoms, often protein-protein interactions are commanded by a relatively small number of interfacial amino acid residues that are part of short linear recognition motifs (London et al., 2010). Standard drug design approaches employed for the development of small molecule inhibitors have been found generally insufficient compared to peptide constructs in disrupting protein-protein interactions. Peptides, being larger than most small molecule inhibitors, can target large and shallow binding sites with a small ratio of binding energy per atom (Kastritis and Bonvin, 2013), which are often categorized as “undruggable” by standard definition.

A large variety of approaches are available to model small molecule-protein interactions. These range from ligand-based cheminformatic and pharmacophore models (Lavecchia and Di Giovanni, 2013; Yan et al., 2016), to structure-based docking and scoring (Ewing et al., 2001; Gray et al., 2003; Verdonk et al., 2003; Friesner et al., 2004; Kozakov et al., 2006; Zhou et al., 2007; Perryman et al., 2014; Pierce et al., 2014) and physics-based atomistic models (Gilson et al., 1997; Wang et al., 2015; Bell et al., 2016; Ellis et al., 2016; Zuckerman and Chong, 2017). Which are the focus of this work. The ultimate goal of physics-based computational models of binding is the quantitative prediction of equilibrium constants of binding (or, equivalently, standard free energies of binding) from statistical mechanics principles and models of interatomic interactions (Chang et al., 2007; Gallicchio and Levy, 2011). Pathway-based models of binding measure the free energy changes along a thermodynamic path linking the unbound and bound states of the molecular complex. These take the form of physical pathways in which ligand and receptor are brought together along a spatial coordinate (Hénin et al., 2005; Gumbart et al., 2013; Jo et al., 2015; Lapelosa, 2017), as well as pathways in so-called alchemical space in which ligand-receptor interactions are progressively dialed-in (Chodera et al., 2011; Deng et al., 2017).

Models targeting protein-peptide binding are generally not as established (Kilburg and Gallicchio, 2016). Bioinformatic and knowledge-based approaches based, for example, on sequence and structure-based homology and phylogenetic profiles, are commonly employed to search for likely interacting protein pairs (Shoemaker and Panchenko, 2007; Zhang et al., 2012). The ability of predicting binding affinities of protein-peptide interactions from structural models is a crucial challenge that would enhance our understanding of biological regulatory systems and be highly beneficial for drug design and development. Even though estimating binding free energies from physical principles is a daunting computational problem, worthwhile attempts should be made to solve it. A main obstacle is that the size and flexibility of peptides make them difficult systems to model (You et al., 2016). Following the work of characterizing physical pathways approaches for this problem (Hénin et al., 2005; Gumbart et al., 2013; Jo et al., 2015; Kilburg and Gallicchio, 2016; Lapelosa, 2017), the purpose of this work is to assess the applicability of alchemical methods for the estimation of protein-peptide binding free energies.

The double-decoupling method (Gilson et al., 1997), is the leading approach for the alchemical calculation of absolute binding free energies from first principles. It is based on a thermodynamic cycle involving the free energies of decoupling the ligand from the solution with and without the presence of the receptor. One of the difficulties of this approach is that the binding free energy, which in reversible binding processes is relatively small and weakly dependent on ligand size, is obtained as the difference of two much larger values which grow with ligand size and ligand charge and whose statistical and systematic errors combine additively (Deng and Roux, 2009). For example, for a protein-peptide complex, the double-decoupling approach would require the challenging calculation of the large solvation free energy of the peptide within an uncertainty small in comparison to the binding free energy (ideally a fraction of a kilocalorie per mole). Furthermore, the free energy of decoupling from the receptor environment, which arguably involves much slower and complex reorganization processes than the decoupling from the solution, would have to be converged to the same degree (Gumbart et al., 2012).

To begin to address these challenges, in this work we assess the applicability of a single-decoupling alchemical method (Gallicchio et al., 2010) (referred to hereafter as “SDM” for short) to the calculation of protein-peptide binding free energies. As outlined below, the method employs a λ-dependent potential energy function which interpolates between the dissociated and associated states of the complex in solution, avoiding the intermediate gas-phase state required by the double-decoupling method. Because it computes the binding free energy directly, the method is expected to exhibit favorable convergence properties sufficient to handle challenging protein-peptide systems. Indeed the method has been already tested with relatively large ligands including some to the same receptor site (discussed below) which is the object of this work (Lapelosa et al., 2012; Gallicchio et al., 2014).

HIV-1 is a RNA retrovirus that must integrate a reverse transcribed copy of its RNA genome into host DNA to permanently infect a cell (Smith and Daniel, 2006). Essential viral catalytic enzymes include reverse transcriptase (RT) and integrase (IN). The former converts the viral RNA genome into double stranded cDNA. Once inside the nucleus, IN catalyzes the insertion of viral cDNA into the host chromosome (Cherepanov et al., 2005a). Integrase catalyzes this reaction in two ways: (1) it removes the 3′ terminal GT nucleotides from both ends of the viral cDNA and (2) inserts the newly processed 3′ termini into host chromosomal DNA (Cherepanov et al., 2005b). However, HIV1-IN itself cannot associate with chromatin and requires an endogenous transcriptional coactivator, p75, more commonly known as LEDGF (lens epithelium-derived growth factor) (Cherepanov et al., 2005b). In recent years, several small molecule inhibitors of the HIV1-IN interaction with LEDGF have been designed by focusing on interactions with selected residues of LEDGF, most notably Ile365, Asp366, and Leu368 (Cherepanov et al., 2005b; Rhodes et al., 2011). However, known mutations of HIV1-integrase have shown high resistance to these inhibitors, prompting further study into other potential interactions between LEDGF and integrase (Rhodes et al., 2011).

**Figure 1:**
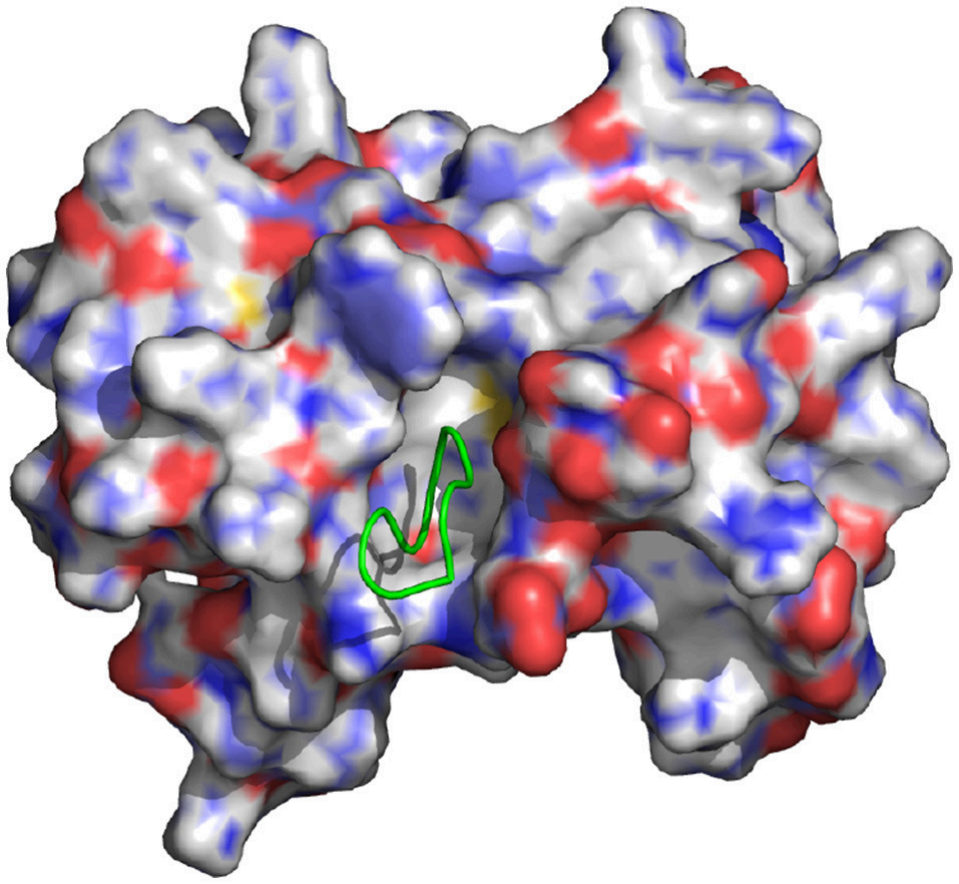
Representation of the wild-type cyclic peptide (SLKIDNLD) bound to the LEDGF binding domain of HIV1 integrase (PDB id: 3AVB). The cyclic peptide (green) is shown in tube representation. The integrase receptor is shown in surface representation colored by electrostatic charge.

An extensive study by Tsiang et al. (2009) reported the binding affinities of native LEDGF to HIV1-IN as well as many LEDGF derived peptides, including both linear and cyclized motifs. One of the goals of their study was to find the smallest LEDGF-peptide sequence capable of inhibiting integrase. They found that small peptides are indeed capable of displacing LEDGF from HIV1-IN and concluded that cyclizing the peptides resulted in greater potency. Building on this, Rhodes et al. (2011) studied the interactions between HIV1-IN and multiple linear and cyclic peptides derived from the SLKIDNLD (residues 362-369) binding motif of LEDGF (Figure 1). This motif is located on a loop region, between the α1 and α2 helices of LEDGF, which is considered the main interaction site (Cherepanov et al., 2005b). Rhodes et al. found that, in agreement with previous studies (Tsiang et al., 2009), the linear peptide, H-SLKIDNLD-OH, gave no activity up to 1 mM, while a cyclized version of the same sequence produced an IC_50_ value of 70.0 μM using a strand transfer inhibition assay.

In this work we conduct alchemical single-decoupling calculations of the binding free energies of a set of LEDGF-derived peptides to the HIV1-IN receptor. The results recapitulate the effect of mutations relative to the wild-type binding motif of LEDGF, particularly those at positions that have been found to be critical for binding, and they provide structural, energetic, and dynamical interpretations of the observed binding affinities. The equilibration of the systems and the convergence of the results are carefully analyzed. The method is found to yield reliable binding free energies of cyclic peptides while it fails to produce a converged estimate for a linear peptide. We discuss the SDM methodology and the procedure of choosing appropriate simulation parameters. We also discuss some of the challenges and potential pitfalls of binding free energy calculations of complex molecular systems which have generally limited their applicability to the quantitative study of protein-peptide binding equilibria.

## 2. Methods

### 2.1. System preparation

The LEDGF binding site construct of HIV integrase was prepared as previously reported (Gallicchio et al., 2014). Water molecules, bound ligands, and crystallization ions were removed and protein sidechain protonation states were assigned assuming pH 7 and absence of significant pKa shifts (Glu and Asp deprotonated and Lys and Arg protonated). A key residue in the LEDGF binding site, His171, was protonated at the Nδ position as previously investigated (Gallicchio et al., 2014). The cyclic SLKIDNLD peptide was adapted from the crystal structure of HIV integrase from which it was bound, 3AVB, as formerly reported (Rhodes et al., 2011). Four cyclic mutants (ALKIDNLD, ALKIDNMD, SLKINNLD, and SLKADNLD) in addition with a linear peptide H-SLKIDNLD-OH were constructed by modifying the cyclic SLKIDNLD peptide using the Maestro program (Schrödinger, LLC).

### 2.2. Single-decoupling binding free energy protocol

Absolute binding free energies of the HIV-IN with the cyclic and linear H-SLKIDNLD-OH peptide complexes were computed with implicit solvation using the Single Decoupling (SDM) (Gallicchio et al., 2010) version of Double Decoupling Method (DDM) (Gilson et al., 1997). SDM was originally introduced as the Binding Energy Distribution Analysis Method (BEDAM) (Gallicchio et al., 2010) when it was based on the Weighted Analysis Histogram Method (WHAM) (Kumar et al., 1992). Recent applications of SDM have been based on multi-state bin-less free energy inference methods (Shirts and Chodera, 2008; Tan et al., 2012). Similarly to double-decoupling, in SDM the standard binding free energy $\Delta G_{\text{b}}^{◦}$ between a receptor R and the ligand L is expressed as:

(1) $$\begin{array}{r} \Delta G_{\text{b}}^{◦}=-k_{B}T\ln\;C^{◦}V_{\text{site}}+\Delta G_{\text{b}} \end{array}$$

where *C*^◦^ is the standard concentration of ligand molecules (1M or 1,668 Å^−3^), *V*_site_ is the volume of the binding site (see below), and Δ*G*_b_ is the excess binding free energy, defined as the free energy difference between the coupled state, in which the receptor and ligand are fully interacting, and the uncoupled state, in which the receptor and ligand are only interacting with the solvent and not with each other. In both the coupled and uncoupled states the ligand is sequestered in the binding site region as defined below.

Unlike the Double-Decoupling Method (DDM), which requires two alchemical calculations going through an intermediate “vacuum” state (Gilson et al., 1997; Boresch et al., 2003), SDM employs a λ/temperature-dependent reduced effective potential energy function which allows for a direct alchemical thermodynamic path between the uncoupled and coupled states of the complex, as follows:

(2) $$\begin{array}{r} U_{\lambda }\left(\text{r}\right)=\beta \left[U_{0}\left(\text{r}\right)+\lambda u\left(\text{r}\right)\right], \end{array}$$

where β = 1/*k*_*B*_*T*,

(3) $$\begin{array}{r} U_{0}\left(\text{r}\right)=U_{R}\left({\text{r}}_{R}\right)+U_{L}\left({\text{r}}_{L}\right) \end{array}$$

is the effective potential energy function corresponding to the uncoupled state, and

(4) $$\begin{array}{r} u\left({\text{r}}_{R},{\text{r}}_{L}\right)=U\left({\text{r}}_{R},{\text{r}}_{L}\right)-U\left({\text{r}}_{R}\right)-U\left({\text{r}}_{L}\right) \end{array}$$

is the binding energy function defined as the effective potential energy difference between the coupled and uncoupled states of the complex in conformation *r* = (*r*_*r*_, *r*_*L*_), obtained by translating the ligand from the solvent and into the receptor without changing the internal coordinates of either molecule. It is straightforward to show that the λ-dependent potential energy function in Equation (2) linearly interpolates between the uncoupled and coupled states of the complex as λ is varied from 0 to 1.

In lieu of simulating each λ and/or temperature state independently and to improve conformational sampling efficiency, with SDM we utilize a Hamiltonian replica exchange (HREM) λ/temperature-hopping approach where λ and temperature values are swapped periodically according to a Monte Carlo procedure with acceptable exchanges adhering to the Metropolis algorithm (Pal et al., 2016).

To further improve convergence of the free energy at values around λ = 0, we apply in this work a “soft core” binding energy function:

(5) $$u^{\prime }(r)=\{\begin{array}{ll} {\text{u}}_{\text{max}}\tanh\left(\frac{\text{u}(r)}{{\text{u}}_{\text{max}}}\right) & \text{if u}(r)>0\\ \text{u}(r) & \text{if u}(r)\leq 0 \end{array}$$

where *u*_max_ is some large positive value compared to the thermal energy. In this work we have set *u*_max_ = 1000 kcal/mol. The energy function modeled in Equation (5) replaces the binding energy function in Equation (4), when applicable, by limiting the maximum value of the binding energy for very small λ values while leaving the values of the favorable binding energies unchanged. The resulting data is subsequently processed using UWHAM analysis (Tan et al., 2012) to calculate the binding free energy of the complex.

The reorganization binding free energy is calculated by taking the difference between the binding free energy and the average interaction energy as:

(6) $$\begin{array}{r} \Delta G_{\text{reorg}}^{◦}=\Delta G_{b}^{◦}-\Delta E_{b} \end{array}$$

Additionally, an advantage of calculating the binding free energy multiple temperatures is the availability of the conformational entropy of binding $\Delta S_{b}^{◦}$

(7) $$\begin{array}{r} \Delta S_{b}^{◦}=-\frac{\partial }{\partial T}\Delta G_{b}^{◦} \end{array}$$

As we have only several binding free energies at discrete temperatures, the above differential was solved by using a linear least squares approximation.

(8) $$\begin{array}{r} f=\underset{i}{\sum }{\left(\frac{b+mx_{i}-y_{i}}{\sigma_{i}}\right)}^{2} \end{array}$$

(9) $$\begin{array}{r} \frac{\partial f}{\partial m}=\underset{i}{\sum }\left(\frac{\left(b+mx_{i}-y_{i}\right)x_{i}}{\sigma_{i}^{2}}\right)=0 \end{array}$$

and

(10) $$\frac{\partial f}{\partial b}=\sum_{i}\left(\frac{(b+mx_{i}-y_{i}}{\sigma_{i}^{2}}\right)=0$$

Solving these two simultaneous equations yields

(11) $$\begin{array}{r} \Delta S_{b}^{◦}=\frac{\sum \left(1/\sigma_{i}^{2}\right)\sum \left(x_{i}y_{i}/\sigma_{i}^{2}\right)-\sum \left(x_{i}/\sigma_{i}^{2}\right)\sum \left(y_{i}/\sigma_{i}^{2}\right)}{\sum \left(1/\sigma_{i}^{2}\right)\sum \left(x_{i}^{2}/\sigma_{i}^{2}\right)-{\left(\sum x_{i}/\sigma_{i}^{2}\right)}^{2}} \end{array}$$

The error on the entropy was calculated using the errors on the individual points.

(12) $$\begin{array}{r} \frac{1}{\sigma^{2}}=\sum \left(\frac{{\left(x_{i}-<x>\right)}^{2}}{\sigma_{i}^{2}}\right) \end{array}$$

Finally, the binding reorganization energy Δ*E*_reorg_ was computed as the residual of the reorganization free energy of binding after subtraction of the entropic component:

(13) $$\begin{array}{r} \Delta E_{\text{reorg}}=\Delta G_{\text{reorg}}+T\Delta S_{b}^{◦} \end{array}$$

The errors reported for: Δ*E*_reorg_ and Δ*G*_reorg_ were calculated using standard error propagation. For Δ*E*_*b*_ we used the standard error of the mean. All statistical uncertainties, including error bars in plots, are reported as twice the standard deviation (96% confidence interval).

### 2.3. Computational details

In this work we employ an effective potential energy function based on the AGBNP2 implicit solvent model (Gallicchio and Levy, 2004; Gallicchio et al., 2009), and the OPLS-AA force field (Jorgensen et al., 1996; Kaminski et al., 2001) for the covalent and non-covalent interactions. Parallel molecular dynamics simulations were performed with the IMPACT program (Banks et al., 2005). Replica exchange conformational sampling was conducted for all combinations of eight temperature spanning 300 to 379 K, and 26 intermediate λ steps at λ = 0.0, 0.002, 0.0048, 0.006, 0.008, 0.01, 0.015, 0.02, 0.0225, 0.025, 0.03, 0.0325, 0.035, 0.04, 0.07, 0.1, 0.25, 0.35, 0.45, 0.55, 0.65, 0.71, 0.78, 0.85, 0.92, and 1, for a total of 208 replicas. The binding site volume was defined as any conformation in which the peptide center of mass is within 6 Å of the center of mass of the C_α_ atoms of residues 168-174 and 178 of chain A and residues 95-99, 102, 125, 128, 129, and 132 of chain B (residue and chain designations according to 3NFB crystal structure) of HIV1-IN. The peptide was sequestered within the binding site by means of a flat-bottom harmonic potential with a force constant of 3.0 kcal/mol Å^2^ applied to atoms with distances greater than 6 Å. The volume of the binding site is calculated to be 904 Å corresponding to $k_{B}T\ln\;C^{◦}V_{\text{site}}=0.69$ kcal/mol. As in previous work (Gallicchio et al., 2014), the C_α_ atoms of the residues of the CCD domain of HIV1-IN were restrained to the overall structure by means of spherical harmonic restraints with a force constant of 1 kcal/mol Å^2^, excluding the residues closest to the binding site (residues 167-178 of chain A and 95-102 and 124-132 of chain B) which were left unrestrained.

We simulated the complexes of HIV1-IN with six peptides: cyclic SLKIDNLD, linear H-SLKIDNKL-OH, cyclic ALKIDNLD, cyclic ALKIDNMD, cyclic SLKINNLD, and cyclic SLKADNLD. Multi-dimensional replica exchange calculations were performed for about 3.3 ns of molecular dynamics per replica, or 867 ns of average simulation time for each complex and approximately 5.2 μs of simulation time in total. Binding energies were sampled with the frequency of 25 ps for a total of 35,000 binding energy samples per complex. All binding free energy calculations were conducted on Louisiana State University's XSEDE SuperMic supercomputer using the ASyncRE job distribution middleware (Gallicchio et al., 2015).

### 2.4. Error analysis and determination of equilibration and convergence

Statistical uncertainties of binding free energy estimates were calculated using UWHAM's built-in Fisher's analysis. The UWHAM formalism, based on mathematical distribution theory, uses the curvature of the likelihood function to calculate the variance of free energy estimates. A detailed derivation is provided by Tan et al. (2012) Fisher's analysis assumes uncorrelated data, we therefore assessed the statistical inefficiency of the binding energy time series data by computing uncertainties based on a standard $1/\sqrt{N}$ error on progressively smaller, random sets of the data and then subsequently plotted those errors against the error provided by UWHAM that contained the same number of data points. From these comparisons, as well as the data obtained from running auto-correlation analysis, we have concluded that binding energies were collected with sufficiently small frequency so as to make statistical correlations negligible.

**Figure 2:**
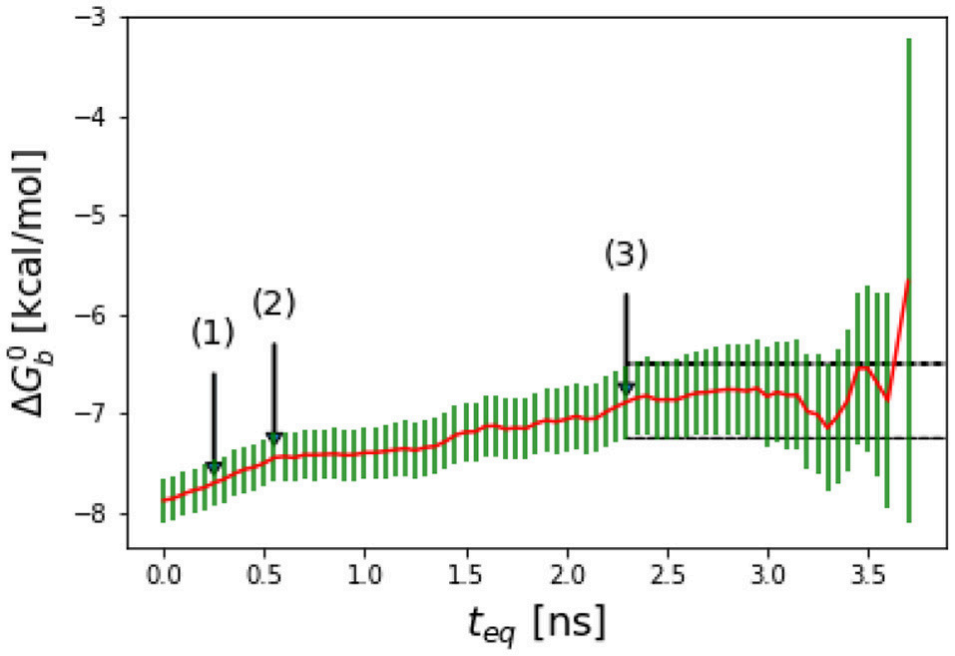
Representative reverse cumulative convergence plot for the binding free energy for the complex with peptide Mutant 2 (S362A/L368M) highlighting the different methods for choosing equilibration time. (1) represents the equilibration time, *t*_eq_, returned by the quantitative protocol we adopted in this work as described in the text, (2) represents the *t*_eq_ chosen qualitatively based on the first observed inflection point of the plot and (3) represents the smallest *t*_eq_ corresponding to a free energy estimate statistically indistinguishable from those at longer equilibration times. The resultant $\Delta G_{b}^{0}\left(t_{eq}\right)$ are −7.7 ± 0.2, −7.5 ± 0.2, and −7.1 ± 0.4 kcal/mol with the three methods, respectively. The three methods yield statistically equivalent results in this case.

Most molecular dynamics simulations are initiated with structures that are atypical of equilibrium conformations. As binding energy calculations are sensitive to small perturbations in configuration, it is typical practice to remove an initial portion of the trajectory in which the system is approaching equilibration so as not to adulterate the equilibrium result (Klimovich et al., 2015). To determine the amount of initial data to eliminate, in this work we employ a method similar in the spirit of reverse cumulative averaging from Yang and Karplus (2003) and the autocorrelation analysis discussed recently by Chodera (2016) In this approach, we examine the time series of binding free energy estimates as a function of increasing equilibration time *t*_eq_ measured from the beginning of the simulation. Specifically, we define $\Delta G_{b}^{◦}\left[t_{\text{eq}}\right]$ as the binding free energy estimate obtained by discarding initial data up to simulation time *t*_eq_. The sequence of binding free energy estimates so obtained is referred to as the reverse cumulative profile (Yang and Karplus, 2003). In these profiles (see Figure 2 as an example) binding free energy estimates to the right, at long equilibration times, are those least affected by bias introduced by unequilibrated data at early times. Conversely, estimates to the left, at short equilibration times, are expected to be the most biased. The decision of which equilibration time to pick is not obvious because the least biased estimates, since they correspond to the smallest binding energy samples, are also those with the largest statistical uncertainties. A suitable equilibration time can be chosen qualitatively as, for example, the smallest equilibration time that gives a free energy estimate statistically indistinguishable from those at longer equilibration times (see Figure 2) (Yang and Karplus, 2003).

**Figure 3:**
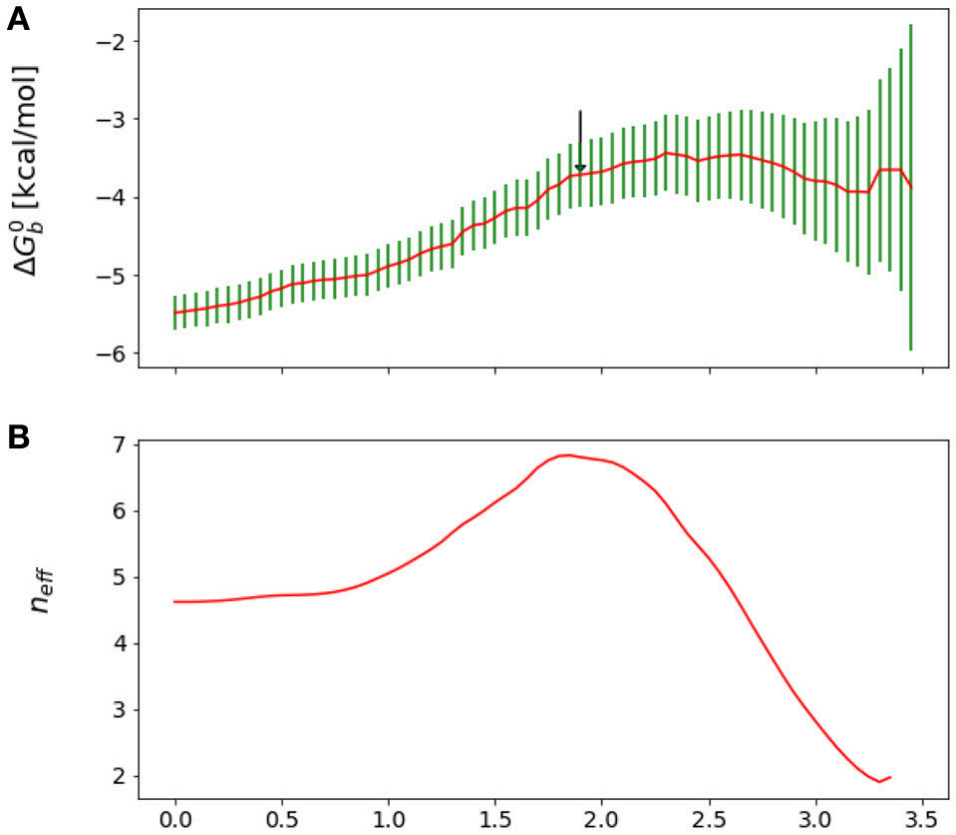
**(A)** Reverse cumulative binding free energy convergence plot for the complex with peptide Mutant 4, I365A. The arrow points to the computed equilibration time. **(B)** Plot of the effective number of independent samples, *n*_eff_, as a function of equilibration time for the same complex. The maximum indicates the estimated equilibration time of 1.9 ns.

In this work we have explored the quantitative protocol for the choice of the equilibration time recently proposed for averages of correlated time series (Chodera, 2016). When applied to a generic time series *x*_*i*_ = *x*(*t*_*i*_), this method consists of picking the equilibration time so as to maximize the effective number, *n*_eff_, of statistically independent samples of the time series (Allen and Tildesley, 1993) which remains after removing the first *n*_*eq*_ data points collected prior to the chosen equilibration time (see Figure 3 for an example). The number of statistically independent samples is given by

(14) $$\begin{array}{r} n_{\text{eff}}=\frac{n-n_{\text{eq}}}{g\left(n_{\text{eq}}\right)}, \end{array}$$

where *n* is the total number of points in the time series, *n* − *n*_eq_ is the number of points remaining in the time series after removing the first *n*_eq_ data points, and

(15) $$\begin{array}{r} g\left(n_{\text{eq}}\right)=1+2\tau_{\text{eq}} \end{array}$$

is the statistically inefficiency of the time series, where τ_eq_ is the correlation length given by:

(16) $$\begin{array}{r} \tau_{\text{eq}}=\overset{t_{max}-1}{\underset{t=1}{\sum }}\left(1-\frac{t}{t_{max}}\right)C_{t} \end{array}$$

where the autocorrelation function, *C*_*t*_ is defined as:

(17) $$\begin{array}{r} C_{t}=\frac{{\langle x_{n}x_{n+t}\rangle -\langle x_{n}\rangle }^{2}}{\langle x_{n}^{2}\rangle -{\langle x_{n}\rangle }^{2}} \end{array}$$

It was found that this approach incorporates the trade-off, discussed above, between minimizing the variance while at the same time minimizing the bias in the estimate. The variance obviously decreases as the number of samples included in the estimate increases, that is as *n*_eq_ decreases. In contrast, Chodera observed that the correlation length, and, consequently, the statistical inefficiency, grow as atypical samples at the beginning of the simulation are included (Chodera, 2016), presumably because these introduce an overall drift in the time series.

We obtained reasonable estimates of equilibration times by applying the approach above to the reverse cumulative profile of the binding free energy $\Delta G_{b}^{◦}\left[t_{\text{eq}}\right]$. We rationalize this observation by noting that, while the reverse cumulative profile is not a time series of an observable, *n*_eff_ still captures the trade-off between maximization of the number of samples and minimization of the statistical inefficiency, which is larger at small equilibration times due the overall drift of the reverse cumulative profile in that region (see Figure 3). Specifically, we considered the family of reverse cumulative free energy profiles $\Delta G_{b}^{◦}\left[t>t_{\text{eq}}\right]$, that is those derived by the full reverse cumulative profile after removal of the values for *t* < *t*_eq_. For each profile we performed autocorrelation analysis to compute the corresponding correlation length τ(*t*_eq_), the statistical inefficiency *g*(*t*_eq_) = 1 + 2τ(*t*_eq_) and effective number of statistically independent samples *n*_eff_ ∝ (*t*_max_ − *t*_eq_)/*g*(*t*_eq_). We then selected the optimal equilibration time as that one that maximizes *n*_eff_. As illustrated in Figure 3, in the case of mutant 4 (I365A), for example, this procedure returns an equilibration time of 1.9 ns per replica.

**Figure 4:**
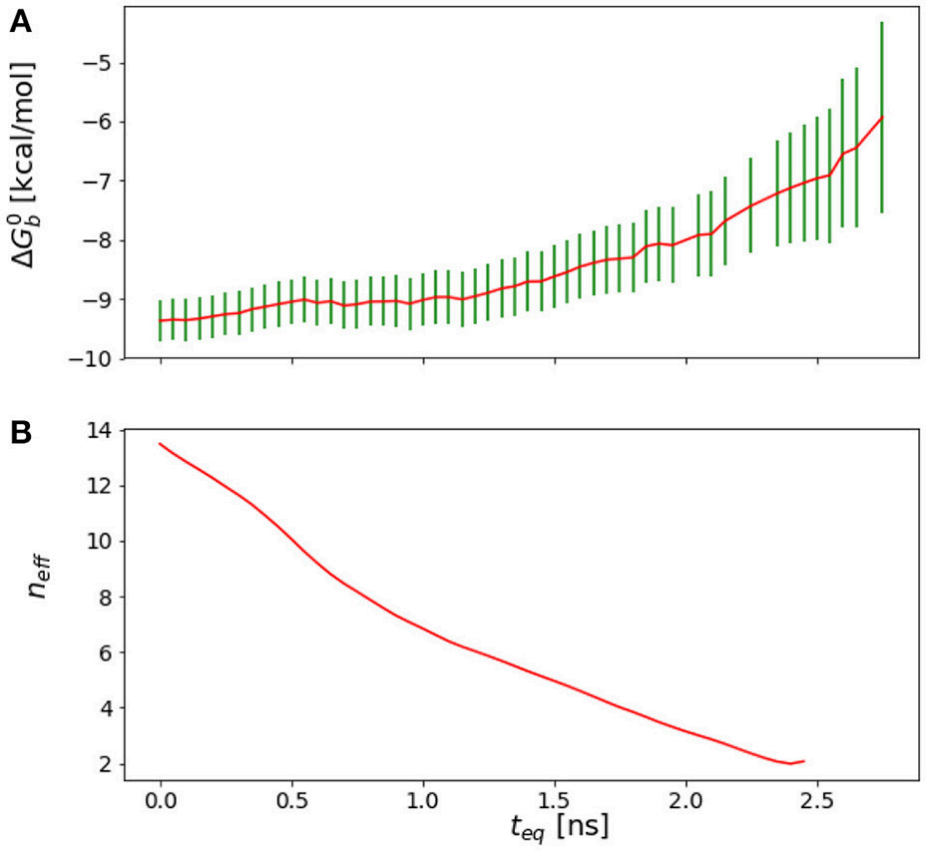
**(A)** Reverse cumulative convergence plot and **(A)** corresponding *n*_*eff*_ plot for the binding free energy of the complex of HIV integrase with the SLKIDNLD linear peptide. Both figures show that neither a qualitative **(A)** nor a quantitative approach **(B)** provides a valid equilibration time for this system. The conclusion is that the binding free energy for this system has not reached equilibration.

In general, we observe that the outlined procedure returns small equilibration times for systems that would be considered to have equilibrated quickly based on qualitative arguments, statistical uncertainties, and the shape of the reverse cumulative profiles. Conversely, the procedure returned relatively long equilibration times (see Results) for systems that qualitatively appear to equilibrate slowly, for example those that display persistent drift of the reverse cumulative profile for short equilibration times (Figures 3, 4). In the case of a linear peptide, considered from multiple perspective to have failed to achieve equilibration, this procedure correctly indicated the lack of a reasonable equilibration time as the *n*_eff_ curve did not display a clear maximum as shown in Figure 4.

**Figure 5:**
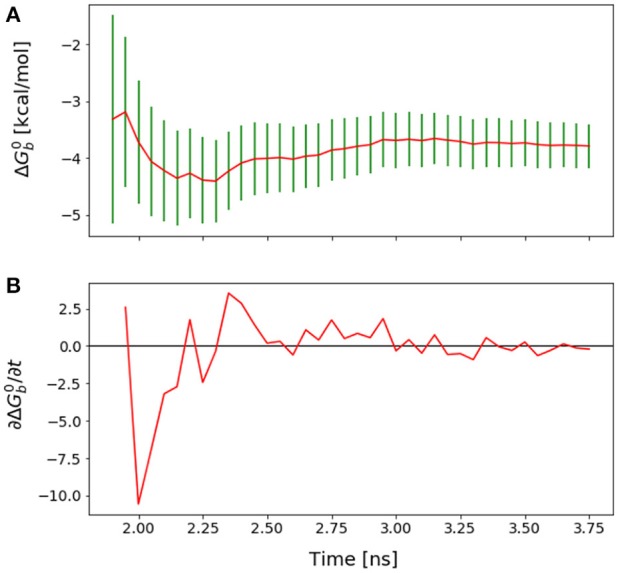
**(A)** Forward cumulative convergence plot for Mutant 4 after the initial bias has been removed. **(B)** Plot of the fluctuations of $\Delta G_{B}^{0}$ that shows a steady decay toward zero.

Having determined the equilibration time, we then assessed convergence of the free energy calculations by examining the conventional forward cumulative profiles (see Figure 5 and Figure S1 for an example) obtained considering only binding energy samples collected at times following the equilibration time until some maximum time *t*, which is varied. Systems that are considered converged display a forward cumulative profile with monotonically decreasing uncertainties, ideally random fluctuations, or, more often, small downward or upward drifts contained within computed uncertainties. To better assess convergence, it has also been helpful to consider the quantity $\left(\delta \Delta G_{b}^{0}/\delta t\right)$, which measures the differential effect of adding samples contained between *t* and *t* + Δ*t*. As shown for example in Figure 5, in converged calculations this function progressively decays to zero with smaller and smaller fluctuations with increasing simulation time.

## 3. Results

### 3.1. Thermodynamic parameters

Computed thermodynamic parameters for the binding of five cyclic LEDGF-derived peptides considered in this work are listed in Table 1. The complexes with wildtype (SLKIDNLD), mutant 1 (ALKIDNLD), and double-mutant 2 (ALKIDNMD) have been studied by Rhodes et al. (2011) who obtained *IC*_50_ values and crystal structures. Mutants 3 (SLKINNLN) and 4 (SLKADNLD) have been included in this study to probe the thermodynamic and structural consequences of mutations in residues known to be critical for successful binding (Tsiang et al., 2009). We have been unsuccessful at computing the binding free energy of the linear wild type peptide (see below), which showed weak or no activity in experimental tests (Tsiang et al., 2009; Rhodes et al., 2011).

**Table 1:** Computed thermodynamic parameters for the binding between HIV1-integrase and a series of cyclic peptides derived from the LEDGF protein.

**Peptide**	**$\Delta G_{b}^{◦}$*^a^***	***ΔE*_*b*_*^a^***	***ΔG*_reorg_*^a^***	***ΔE*_reorg_*^a^***	**$T\Delta S_{b}^{◦}$*^a^***
SLKIDNLD (WT)	−8.6 ± 0.4	−59.3 ± 0.5	50.6 ± 0.5	23.6 ± 1.9	27.0 ± 1.8
ALKIDNLD (M1)	−8.1 ± 0.2	−56.9 ± 0.4	48.8 ± 0.4	22.1 ± 0.4	26.7 ± 0.1
ALKIDNMD (M2)	−7.6 ± 0.2	−59.5 ± 0.5	51.9 ± 0.5	24.6 ± 0.8	27.3 ± 0.6
SLKINNLD (M3)	2.7 ± 0.2	−36.1 ± 0.6	38.3 ± 0.6	12.4 ± 0.6	25.9 ± 0.6
SLKADNLD (M4)	−3.7 ± 0.2	−57.6 ± 0.5	53.9 ± 0.5	33.2 ± 0.6	20.7 ± 0.4

a *In kcal/mol*.

**Figure 6:**
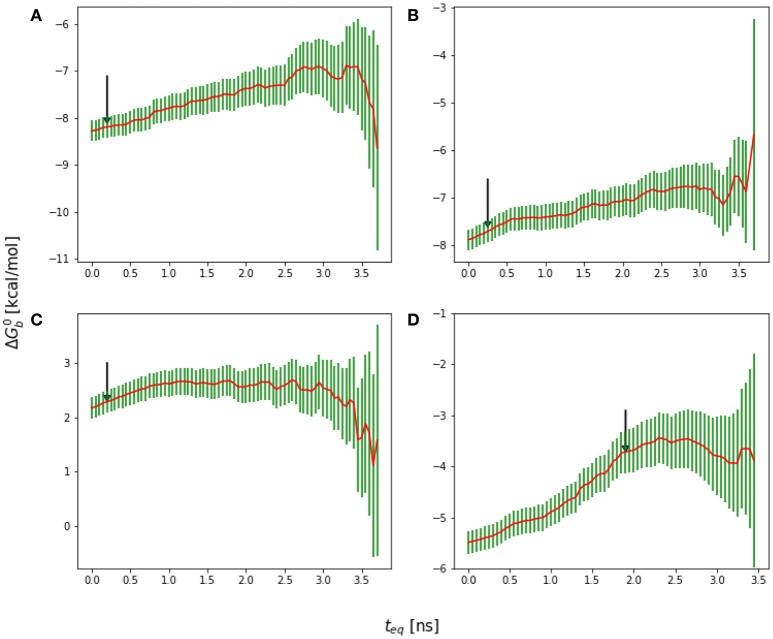
Reverse cumulative binding free energy convergence plots for **(A)** Mutant 1 (S362A), **(B)** Mutant 2 (S362A/L368M), **(C)** Mutant 3 (D366N), and **(D)** Mutant4 (I365A). Arrows mark the estimated equilibration times based on auto correlation analysis: **(A)** 0.20 ns, **(B)** 0.25 ns, **(C)** 0.20 ns, and **(D)** 1.90 ns.

**Figure 7:**
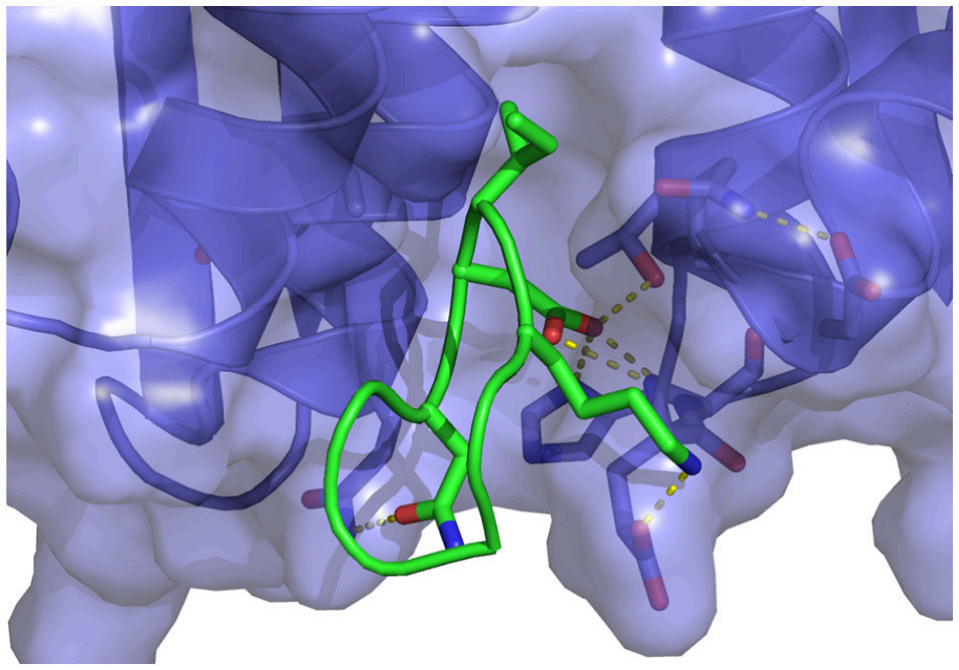
Representative structure extracted from the simulation of the wildtype cyclic peptide (WT in Table 1) bound to the LEDGF-binding domain of HIV integrase. The following intermolecular interactions are highlighted: Lys364-Glu170, Asp366-Thr174, His171, and Asn367-Gln95. Also shown in the intramolecular interaction between Asp168 and Gln169 of the peptide and the key bidentated interaction between Asp366 and the backbone nitrogen atoms of Glu170 and His171.

**Figure 8:**
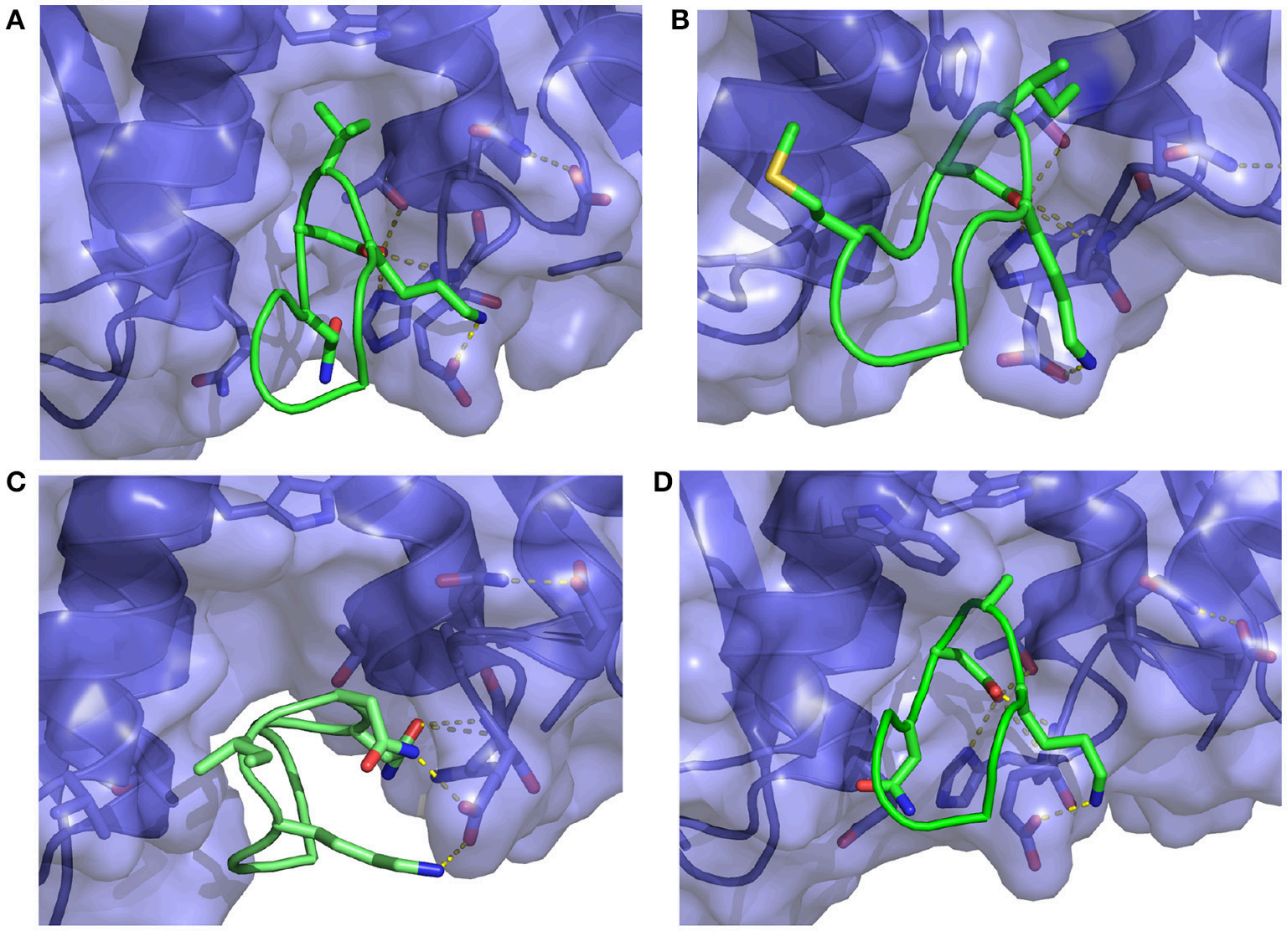
Representative structures extracted from the simulations of **(A)** the S362A mutant cyclic peptide (M1 in Table 1) and **(B)** the S362A:L368M double mutant cyclic peptide (M2), **(C)** the D366N cyclic peptide (M3), and **(D)** the I365A mutant cyclic peptide (M4) with the HIV integrase LEDGF-binding domain. All interactions shown were previously described in Figure 7. Notice in **(C)** that the D366N mutation causes severe distortion of the peptide as well as the receptor relative to the bound conformation of the complex.

The computed binding free energies for wildtype and mutant 1 cyclic peptides are qualitatively consistent with μM-range *IC*_50_ values (Rhodes et al., 2011) measured for these peptides (85.2 ± 24.5 μM and 39.7 ± 7.1 μM, respectively). Also in agreement with inhibition experiments, mutation of the S366 serine residue to alanine causes minor changes in binding affinity. The double mutant S366A/L368M (M2 in Table 1), while undergoing a greater conformational change relative to wild type and mutant 1 (**Figures 7**, **8**), is predicted to have similar binding affinity to the wild type and the single mutant (Figure 6B). Experimental values for this double-mutant are contradictory. Rhodes et al. (2011) report no detectable inhibition activity for this peptide, while they were able to obtain a crystal structure of it bound to HIV integrase (PDB id 3AVJ) (Rhodes et al., 2011). Taken together, this evidence suggests that, while mutant 2 (M2) is capable of binding to HIV integrase, somehow it can not inhibit it effectively.

Consistent with experimental findings (Tsiang et al., 2009; Rhodes et al., 2011), mutation of the critical D366 residue (M3 in Table 1) resulted in a significant reduction of binding affinity. This residue is known to form a strong hydrogen bonding interaction motif with the backbone atoms of His171 and Glu170 of HIV integrase (**Figure 8**), which is also found in many small-molecule inhibitors (Gallicchio et al., 2014). The loss of this interaction is observed to lead to major disruption of the peptide-receptor binding interface and loss of binding affinity. Analogously, mutation of I365 to alanine (M4 in Table 1) causes disruption of favorable hydrophobic packing peptide-receptor interactions and loss of binding affinity, although to a smaller degree than the D366N mutation.

Table 1 also reports the decomposition of the computed binding free energies into average binding energies and reorganization free energies, and the further decomposition of the latter into reorganization energy and conformational entropy of binding. Average binding energies (Δ*E*_*b*_, third column in Table 1) reflect the strength of direct interatomic interactions between the peptide and the protein receptor. These are relatively constant around −58 kcal/mol across the peptide set, with the exception of mutant 3, for which Δ*E*_*b*_ is about 20 kcal/mol less favorable than the others. This behavior confirms that the loss of binding affinity for mutant 3 is mainly due to the loss of key hydrogen bonding interactions between D366 and a histidine residue in the binding pocket of the receptor (**Figure 8**). The magnitude of the interaction energy loss (20 kcal/mol) is also consistent with the loss of two strong hydrogen bonds and the corresponding favorable electrostatic interactions.

In contrast, the weakening of the binding of mutant 4 (M4 in Table 1), in which a bulky isoleucine residue is replaced by alanine, displays a completely different thermodynamic signature. The average binding energy for this peptide complex is of similar magnitude than the higher affinity peptides, and it is actually predicted to be greater than that of mutant 1 even though their predicted binding constants differ by at least two orders of magnitude. The weaker binding of mutant 4 is predicted to be mainly due to a large unfavorable reorganization free energy (fourth column in Table 1), which, in turn, is caused by unusually large intramolecular energy strain (Δ*E*_reorg_) only partially compensated by a smaller entropic penalty ($T\Delta S_{b}^{0}$). As further discussed below, this behavior is consistent with the observed structural response of the peptide and receptor attempting to compensate for the loss of favorable packing interactions (**Figure 8D**). In contrast, the thermodynamic parameters collected in Table 1 indicate that the loss of hydrogen bonding interactions in mutant 3 results, effectively, in the unbinding of the peptide with little or no tendency for conformational reorganization aimed at regaining binding as shown in **Figure 8C** (see below for a discussion of the observed structural changes).

### 3.2. Structural considerations

**Figure 9:**
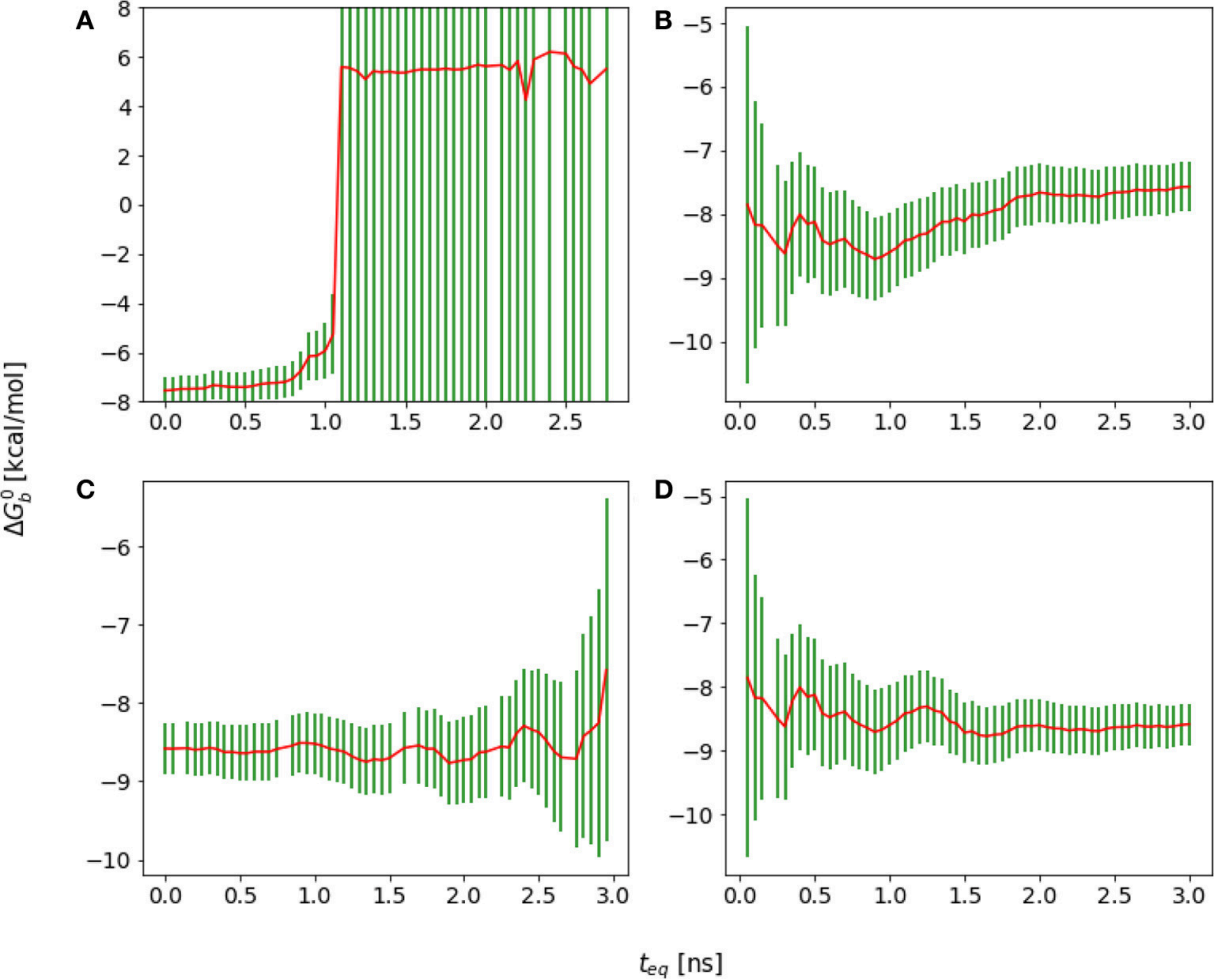
Cumulative plots for the wildtype cyclic peptide. **(A)** Reverse cumulative and **(B)** forward cumulative plots obtained with the original smaller lambda schedule. **(C)** Reverse cumulative and **(D)** forward cumulative plots of the data with the improved lambda schedule. Notice that when there is no equilibrium bias the forward and reverse plots are near mirror images.

The equilibration times of the complexes are found to be highly correlated with the extent of their conformational reorganization to go from the starting structure (which in all cases was the crystal structure of the wildtype peptide) to the equilibrated conformational ensemble. Thus, as expected, the binding free energy of the wildtype peptide converged relatively quickly (**Figure 9B**). Conversely, the I365A mutation caused extensive conformational rearrangements which required a longer equilibration (Figure 6D).

Consistent with the crystal structure (3AVB), residues Ser362, Lys364, Ile365, Asp366, and Asn367 of the cyclic WT peptide form the primary intermolecular interactions with the receptor (Figure 7). The carboxylate group of Asp366 of the peptide forms the strong hydrogen bonds with the NH backbone groups of Glu170 and His171 of the receptor which are the hallmark of the LEDGF-HIV IN complex (Cherepanov et al., 2005a) as well as those with peptido-mimetic synthetic inhibitors (Peat et al., 2014). The carboxylate group of Asp366 is further anchored by an hydrogen bond with the hydroxyl group of Thr174 and with a salt bridge with the protonated nitrogen atom of His171. Both Ser362 and Lys364 of the peptide forms strong interactions with the sidechain of Glu170. Ile365 forms backbone-backbone interactions with Gln168 and Thr174. The sidechain of Ile365 is additionally nestled into an hydrophobic pocket of the receptor lined by residues Ala128, Trp131, Trp132, and Met178. The solvent-exposed residues of the peptide (Leu363, Leu368, and Asp369), do not interact consistently with the receptor although they make occasional intramolecular interactions with each other.

A serine to alanine mutation of residue 362 (M1 mutant) causes a slight, but statistically significant decrease in the predicted binding affinity (Table 1). Auto-correlation analysis of the reverse cumulative binding free energy profiles (Figure 6A) revealed only a small equilibration bias of about 1 kcal/mol, consistent with the relatively minor conformational changes relative to the wildtype peptide (Figure 8A). The most noticeable structural difference is the shift of the interaction between Glu170 and Ser362, now absent, to Asn 367 and the concomitant weakening of the Lys364–Glu170 salt bridge which now occurs less often. These subtle changes are the cause of the weakening of the binding energy and the slight less favorable binding free energy relative to the wildtype (Table 1).

The double mutation S362A:L368M (M2 in Table 1) resulted in no significant change of binding free energy relative to the S362A single-mutant. However, the methionine sidechain at position 368 now affords additional polar interactions with Thr125 and it induces the recruitment of Asp369 (previously completely solvent exposed) in the same interaction (Figure 8B). As a result, the peptide shifts overall position toward the 124-131 α-helix of the receptor.

The D366N mutation resulted, essentially, in the dissociation of the complex (Figure 8C), consistent with the unfavorable computed binding free energy (Table 1, M3 mutant) and previous experiments (Tsiang et al., 2009). The peptide was kept in proximity of the pocket only by the applied tether (see Methods). The conformations of the receptor and the peptide rapidly reorganized to screen the unmet positive charge of His171 while the Asn366 sidechain shifted away from the Glu170 backbone. The rapid equilibration of the peptide, freed from strong intermolecular interactions, is the cause of the smaller equilibration time observed (Figure 6C) for the M3 mutant relative to the other mutants.

The I365A mutant (M4 in Table 1) displayed the most significant and lengthy equilibration of the binding free energy among the peptides studied (Figure 6D). Unlike the other mutants, the most significant structural rearrangement occurred for the receptor. The void in the hydrophobic binding pocket left from the isoleucine to alanine mutation slowly collapsed around the alanine sidechain (Figure 8D). Concomitantly, the peptide moves deeper into the binding cavity causing the solvent-exposed residues (Leu363, Leu368, and Asp369) to make increased interactions with the receptor while slightly decreasing interactions between Asp366 and Glu170/His171. These rearrangements recover most of the interaction energy of binding (Table 1, third column) at the expense of higher conformational strain (fifth column), resulting in a binding free energy 5 kcal/mol less favorable than wildtype in agreement with experimental evidence (Cherepanov et al., 2005b; Tsiang et al., 2009).

### 3.3. The potential pitfalls of protein-peptide binding

Alchemical binding free energy calculations require careful planning and assessment of the results (Klimovich et al., 2015). This is particularly so for protein-peptide complexes due to their large conformational variability. Here we discuss the challenges we encountered in applying our calculation protocols, tuned for small molecule binding, to peptides.

The first challenge we encountered turned out to be related to our initial choice of the λ schedule, which was based on the one that had been used with success on large ligands for the same receptor site (Gallicchio et al., 2014). With this λ schedule we collected a substantial amount of binding free energy data that, when displayed using a traditional forward cumulative plots showed no particular disturbance (Figure 9B) beyond a persistent drift signaling slow convergence. It was only when we analyzed the reverse cumulative plot (Figure 9A) that we discovered that the slow convergence was a symptom of a serious numerical artifact.

The reverse cumulative plot (Figure 9A) shows an abrupt shift in the binding free energy corresponding to an equilibration time of approximately 1 ns. Subsequent examination of the error bounds returned by the UWHAM (Tan et al., 2012) procedure, revealed that without the equilibration data bias introduced by the binding energy data collected at times prior to 1 ns, the uncertainty in the computed binding free energy was so large (on the order of 1000 kcal/mol) as to make the results meaningless.

It was eventually established that the failure in obtaining a reasonable binding free energy estimate was due in this case to the choice of the λ schedule, which lacked sufficient intermediate λ values between 0.1 and 0.4 to ensure sufficient ensemble “overlap” of adjacent alchemical states (Klimovich et al., 2015). Specifically, after some equilibration, for λ < 0.1 all of the binding energy values clustered around the maximum value *u*_max_ [see Equation (5)], which were not observed in any of the other λ states. As a result, the UWHAM multi-state maximum likelihood function (Tan et al., 2012) lacked a well-defined maximum corresponding to the binding free energy profile. This problem had not been apparent when unequilibrated data at the beginning of the simulation, which contained a small amount of binding energy data bridging alchemical states across λ = 0.1, was included in the estimate, resulting in a forward cumulative convergence plot not clearly out of the ordinary.

As a result of this assessment, we modified the λ scheduled to insert intermediate λ states between 0.1 and 0.4 more likely to sample both large and smaller binding energy values. This resulted in binding free energy estimates with reasonable error bounds when analyzed with both forward and reverse cumulative plots (Figures 9C,D).

This experience highlights the need for careful examination of equilibration artifacts and error bounds when assessing the results of alchemical binding free energy calculations. Clearly, in this case, the choices of simulation length and λ schedule tuned for small-molecule binding were not suitable for the modeling of the binding of the larger peptides studied here. The optimized λ schedule addressed the requirement of a suitable, unbroken, thermodynamic path between the coupled and uncoupled states of the complex and enabled the assessment of equilibration bias in the binding free energy estimates. However it did not guarantee convergence of the binding free energy estimates when these are affected by challenges of a different nature, as the following example illustrates.

**Figure 10:**
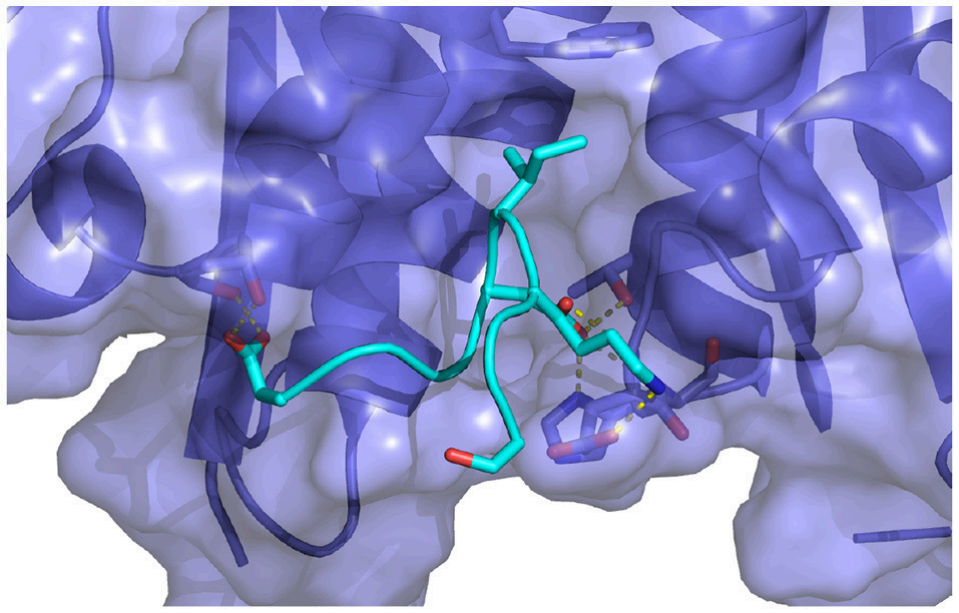
Key interactions observed in the conformational ensemble at low binding energies (around −120 kcal/mol) of the complex of HIV integrase with the linear peptide. Notable differences relative to the complexes with the cyclic peptides include the participation of Asp369 with α_2_ helix residues of HIV integrase.

**Figure 11:**
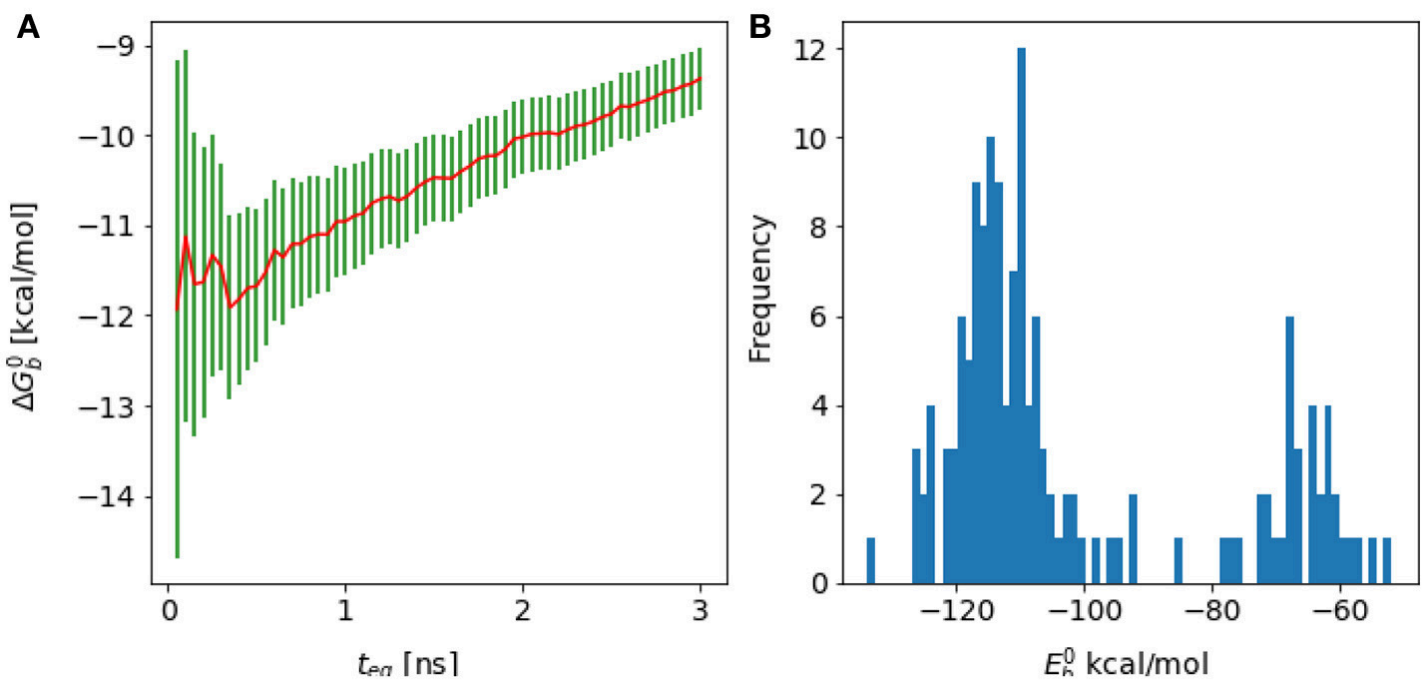
**(A)** Forward cumulative plot and **(B)** histogram of the binding energy distribution at λ = 1 and temperature = 300 K for the complex of HIV integrase with the linear peptide.

One of aims in this work had been to study the thermodynamic factors that favor the binding of the cyclic wild-type peptide over the linear one, H-SKIDNLD-OH (Tsiang et al., 2009; Rhodes et al., 2011) (Figure 10). However, we were unable to obtain a converged binding free energy for the linear form of the wild-type peptide (H-SKIDNLD-OH). As shown in Figure 4, the reverse cumulative plot for the complex with this peptide does not show a plateau, indicating that the system was still in the equilibration phase up until the longest simulation time considered. Lack of convergence is confirmed by the forward cumulative plot including all of the binding energy samples (Figure 11A).

Analysis of molecular dynamics trajectories revealed that the lack of convergence was due to the slow equilibration of the relative populations of two competing conformational states of the complex (Figure 11B). The first state corresponds to a compact conformation of the linear peptide tethered by an intramolecular salt bridge similar to the conformation of the cyclic peptide. The other, more extended, conformation resulted from the replacement of the intramolecular salt bridge with intermolecular salt bridges between the termini of the peptide and residues lying on the rim of the protein receptor pocket (Figure 10). This extended bound conformation, whose formation was found to be reproducible across independent runs, is strongly favored by binding energy (approximately −120 kcal/mol, compared with binding energies of half this magnitude for cyclic peptides and for the compact conformation of the linear peptides–see Table 1 and Figure 11 –and strongly disfavored by reorganization free energy.

Because of the trade-off between energetic and reorganization/entropic opposing thermodynamic driving forces, the compact and extended binding modes were found to coexist at λ = 1, producing a characteristic bimodal binding energy distribution (Figure 11). The binding free energy estimate is found to be very sensitive to the relative population of these two binding modes, which, however, could not be determined with certainty due to lack of interconversion events. As a result, the binding free energy exhibits a slow drift as a function of simulation time with no identifiable convergence point (Figure 4).

Cases, such as this, of slow convergence due to slow intramolecular conformational equilibration can not be easily addressed by conventional conformational sampling acceleration methods based, for example, on Hamiltonian replica exchange on the alchemical variable as done here. Some success has been gained with biasing potentials targeting specific degrees of freedom (Ensing et al., 2006; Kim et al., 2010; Procacci et al., 2013; Cavalli et al., 2014), such as dihedral angles (Wang et al., 2012; Lindert et al., 2013). We have not attempted, in this work, to obtain a converged estimate of the relative population of the two binding modes of the linear peptide using these methods. This is partly because of the awareness that implicit solvation may not be an optimal model for conformational equilibria involving salt bridges (Okur et al., 2008; Gallicchio et al., 2009), even if these could be sampled thoroughly.

## 4. Discussion

Despite decades of intense medical research, HIV infections continue to be a major widespread problem for worldwide health with 37 million people living with AIDS and 21 million on antiretroviral therapy (World Health Organization, 2017). The integrase enzyme of the HIV virus is one of the main targets of ongoing anti-viral treatments. Raltegravir, for example, is an inhibitor of integrase's strand transfer reaction approved for clinical use in 2008. It remains one the most used integrase inhibitors in conjunction with other viral inhibitors in highly active antiretroviral treatment courses (Summa et al., 2008). Even though they are able to significantly reduce viral load, a significant downside of strand transfer integrase inhibitors is that HIV becomes readily resistant to their effects (Murray et al., 2007; Sarkis et al., 2008). There has been therefore great interest in identifying alternative inhibitory pathways for HIV integrase.

The interaction between integrase and LEDGF/p75 is considered one of the most promising untapped targets. LEDGF/p75 is an endogenous cofactor that is essential to viral replication. It facilitates the integration of viral DNA by transporting the viral protein into the cell nucleus and inserting the viral DNA into host DNA. LEDGF/p75 is also believed to play a role in the protection of integrase from degradation by the host cell (Smith and Daniel, 2006). Because it targets a host protein, the LEDGF binding domain of HIV integrase is believed to be less susceptible to the insurgence of resistance mutations. There have been efforts to identify peptido-mimetic compounds capable of disrupting the interaction between HIV integrase and LEDGF/p75 (Gallicchio et al., 2014; Peat et al., 2014).

Cherepanov et al. (2005a) reported in 2005 the crystal structure of the complex between HIV integrase and the integrase binding domain of LEDGF (PDB code: 2B4J). The structure revealed key residues responsible for mediating the interaction. Ile365 of LEDGF/p75 is found into the hydrophobic cavity of the integrase binding site that consists of Leu102, Ala128, Ala129, Trp131, Trp132, Thr174, and Met178. Ile365 of LEDGF also formed a hydrogen bond between its backbone amide and the backbone carbonyl group of Gln168 of integrase. Asp366 of LEDGF makes two-pair hydrogen bonds with Glu170 and His171 of integrase. In addition, a salt-bridge is found between Lys364 of LEDGF and Glu170 of integrase. Mutations of these critical residues weaken the LEDFG/integrase interaction (Tsiang et al., 2009).

Based on these earlier studies, Rhodes et al. (2011) investigated the structures of HIV integrase bound to a series of cyclic peptides including the wild-type sequence SLKIDNLD, corresponding to residues 362-369 of LEDGF/p75, as well as single and double mutants. Rhodes et al. obtained crystal structures for 13 of the complexes they investigated (PDB codes: 3AV9, and 3AVA through 3AVN) (Rhodes et al., 2011). While we did not use the obtained crystal structures as templates for our S362A and S362A:L368M mutants, our resulting structures matched well with those reported by Rhodes et al. (2011) The structure for the wild-type cyclic peptide matched most of the intermolecular interactions found in the structure of the complex with LEDGF/p75.

We simulated the SLKIDNLD, ALKIDNLD, and ALKIDNMD cyclic peptides investigated by Rhodes et al. (2011) to compare directly with their findings. We also simulated two other cyclic peptides with known fatal mutations, D366N and I365A, to compare with earlier experiments (Cherepanov et al., 2005a,b; Tsiang et al., 2009). The results of our calculations recapitulate all of the experimental findings and identify the specific interactions responsible for the binding trends (Figures 7, 8, and Table S1). We were able to confirm that three residues of the peptides, corresponding to Lys364, Ile365, and Asp367 of LEDGF, are responsible for the most critical protein-peptide interactions. Conversely, the residue pairs Leu363-Leu368 and Ser362-Asn367 form the most stable intramolecular interactions within the peptides.

In this study, we used an alchemical approach to compute the binding free energies for the complexes of HIV integrase with one linear peptide and five cyclic peptides derived from LEDGF. A significant portion of the effort was devoted to error and convergence analysis (Shirts, 2013; Klimovich et al., 2015). In normal circumstances, visual inspection of the binding data time series could be sufficient to get a good estimate of equilibration time and convergence. In more complex systems, as in this case, variations of binding free energy estimates over the course of the simulation are slow and gradual and require careful consideration. The onset of equilibration is particularly slight, but not less important, when the starting conformation is close to the equilibrium conformation. To estimate equilibration times, that is the amount of data to neglect at the beginning of the simulation, we employed techniques inspired by previous work (Yang and Karplus, 2003; Chodera, 2016). We tested various approaches and concluded that the process described above based on reverse cumulative averaging gave the most reliable and consistent results. We established that, while failing for the linear peptide, the method yielded robust binding free energy estimates for a series of cyclic peptides.

This work represents a rare successful application of an alchemical binding free energy method to the calculation of converged absolute binding energies of protein-peptide complexes (Kilburg and Gallicchio, 2016). This has been possible by employing multi-dimensional replica exchange conformational sampling and by adopting an implicit model of solvation which speeds up equilibration and convergence by removing the fluctuations of the solvent. Statistical errors are further reduced by designing a direct alchemical path from the unbound to bound states of the complex. This is in contrast to double decoupling strategies (Gilson et al., 1997), necessary with explicit solvation, which require going through an intermediate decoupled “vacuum” state of the ligand. Because the free energy changes and the corresponding errors leading to and from the intermediate decoupled state are generally large, double decoupling estimates are affected by slow convergence (Deng and Roux, 2009), especially for large and charged ligands such as these (Gumbart et al., 2012).

Potential of mean force methods (Hénin et al., 2005; Woo and Roux, 2005; Comer et al., 2014; Sandberg et al., 2015; Casasnovas et al., 2017; Deng et al., 2017) have been applied to protein-peptide binding (Hénin et al., 2005; Gumbart et al., 2013; Jo et al., 2015; Lapelosa, 2017). These circumvent the difficulties of double decoupling alchemical methods by following the direct physical path of the ligand in and out of the receptor. Nevertheless, slow conformational reorganization remains a serious bottleneck in these calculations (Gan and Roux, 2009). In some cases convergence has been achieved only by imposing stiff conformational restraints, which introduce significant systematic bias unless rigorous and time-consuming treatments are applied (Gumbart et al., 2013). With the exception of weak positional restraints on portions of the receptor backbone distant from the peptide binding site and the wide flat-bottom center of mass tether required by alchemical theory (Gilson et al., 1997), in the present simulations the receptor and the peptides were free to explore a wide variety of conformations so as to properly respond to the applied mutations (Figure 8).

The implicit description of the solution environment, while critical to achieving converged results, is likely less accurate than the more established and tested explicit solvent descriptions. Implicit solvation models, based on the rigorous concept of the solvent potential of mean force (Roux and Simonson, 1999), are not intrinsically less accurate than other models. However, because they are asked to model a free energy rather than a potential energy, they are more difficult to design and parametrize than explicit models of solvation (Chakavorty et al., 2016). Some implicit solvent implementations, in particular, are known to overemphasize the formation of salt bridges between protein residues (Okur et al., 2008; Wickstrom et al., 2015).

Here we adopt the AGBNP2 implicit solvent model (Gallicchio et al., 2009) which incorporates short-ranged terms and other features designed specifically to tune intermolecular interactions and, in particular, the occurrence of protein salt bridges as seen in experimental NMR structures and explicit solvent simulations. Despite these steps, it is possible that the long-lived intermolecular electrostatic interactions between the termini of the linear peptide and the protein receptor site and the opposing intramolecular interactions between the termini, which prevented convergence of the binding free energy in that case, are artifacts of the AGBNP2 implicit solvent model and the specific choice of fixed protonation state (Ellis et al., 2016; Harris et al., 2017). The model may overestimate the strength of salt bridges or overestimate the free energy barrier separating them from solvent-separated states. The good quantitative agreement between calculated and experimental binding affinities for the cyclic peptides, which also form salt bridges with the protein receptor, suggests that the latter circumstance is more likely. Thus, it appears that the failure for the linear peptide is caused by the slow interconversion rates between competing ion-paired conformations which prevents the reliable estimation of their relative populations and, ultimately, of their relative contribution to the binding free energy.

Specialized conformational sampling techniques based on collective variables (Zheng et al., 2008; Di Leva et al., 2014) and constant pH molecular dynamics (Mongan et al., 2004; Ellis and Shen, 2015) could be applied here to treat these specific slow degrees of freedom related to salt-bridge formation.

While successful in this circumstance, the methodology we employed here for the estimation of protein-peptide binding is not without hurdles. The choice of λ schedule as well as the temperature ladder of the multi-dimensional replica exchange conformational sampling turned out to be critical for the overall realization of convergence. The larger the ligand the more dense the λ and temperature schedules have to be to form enough critical overlap between alchemical states. Careful monitoring of equilibration bias and analysis of convergence proved essential to ensure the robustness and reproducibility of the results in these difficult systems. Furthermore, due to the larger size of the systems, the large number of replicas, and the slow conformational reorganization as compared to small-molecule inhibitors, these calculations are quite expensive and time consuming. We are currently working to port our SDM implementation to GPUs and parallel processors to leverage their high computational power (Zhang et al., 2017).

These hurdles, however, should not be seen as a deterrent. Free energy modeling of protein-peptide binding is an exciting, emerging field with great potential in biomedical research and drug discovery. We have shown here that our single-decoupling strategy, while not perfect, can be used to calculate converged binding free energies for protein-peptide complexes. Advancements in models, algorithms and computer hardware are progressing steadily and making calculations more efficient and practical.

## 5. Conclusions

In this work we evaluate a single-decoupling alchemical method (SDM) combined with implicit solvation and advanced conformational sampling strategies toward the calculation of the binding free energies of protein-peptide complexes. We report converged binding free energy estimates of a set of cyclic LEDGF-derived peptides to the HIV1-IN receptor. The binding free energy estimates recapitulate the observed effect of mutations relative to the wild-type binding motif. We show that careful error analysis and monitoring of equilibration and convergence is essential to ensure the reliability of the results. For example, the analysis detected the failure of the method for a linear peptide due to conformational trapping. In overall, the results of this work confirm that, together with advanced conformational sampling strategies, accurate solvation models, careful quality control, and careful convergence analysis, the single-decoupling alchemical strategy employed here is a viable approach to the quantitative calculation of the binding free energies of protein-peptide complexes.

## Author contributions

DK and EG jointly designed the research and wrote the paper. DK performed the calculations and the numerical analysis.

### Conflict of interest statement

The authors declare that the research was conducted in the absence of any commercial or financial relationships that could be construed as a potential conflict of interest.
