# Supplementary material for: Assessment of a Single Decoupling Alchemical Approach for the Calculation of the Absolute Binding Free Energies of Protein-Peptide Complexes
Source: Front Mol Biosci. 2018 Mar 8;5:22. doi: 10.3389/fmolb.2018.00022 (PMC5852065; doi:10.3389/fmolb.2018.00022)
Supplement: Supplementary file 1 [file DataSheet1.PDF]

Supporting Information

Assessment of a Single Decoupling Alchemical Approach for the Calculation of the Absolute Binding Free Energies of Protein-peptide Complexes

Denise Kilburg[a,b] and Emilio Gallicchio[a,b,c *]

January 4, 2018

[a]Department of Chemistry, Brooklyn College, 2900 Bedford Avenue, Brooklyn, New York 11210. [b]Ph.D. Program in Chemistry, The Graduate Center of the City University of New York, New York NY 10016. [c]Ph.D. Program in Biochemistry, The Graduate Center of the City University of New York, New York NY 10016

1 Supplementary Information:

- Figure 1-supplementary: Reverse and forward cumulative plots for mutants
- Table 1-supplementary: Full list of receptor-peptide interactions

Reverse and forward cumulative data
Below are the reverse (A, C, E, G) and forward (B, D, F, H) profiles for the the four mutant peptides. The profiles shown have had their initial equilibrium/bias data removed. All systems are considered converged as they display monotonically decreasing uncertainties. 75% of the mutants had very small equilibration times due to near-equilibrium starting structures (M1 and M2) or strong repulsive coulombic forces in the case of M3. M4, I365A, was the only mutant with a substantial equilibration time (1.9 ns) and this is a result of a large non-polar residue, replaced by a smaller non-polar residue causing a slow but energetically consequential rearrangement of the peptide and receptor.

Receptor-peptide interactions
Shown below is a table of the average distances between nitrogen and oxygen atoms of the peptide and receptor for all relevant T=300K and λ=1 trajectories. Only distances of less than 4 Å were considered. There were approximately 200 relevant trajectories per mutant. The percentages shown below in the table represent the % of these 200 trajectories/mutant that contained the specific peptide-receptor interaction (measured by interatomic distances less than 4Å). The table shows that Lys364, Ile365, Asp366 are the dominant interacting peptide residues. The table also shows how the participation of the residues varies in response to the mutation.

*Corresponding author: egallicchio@brooklyn.cuny.edu

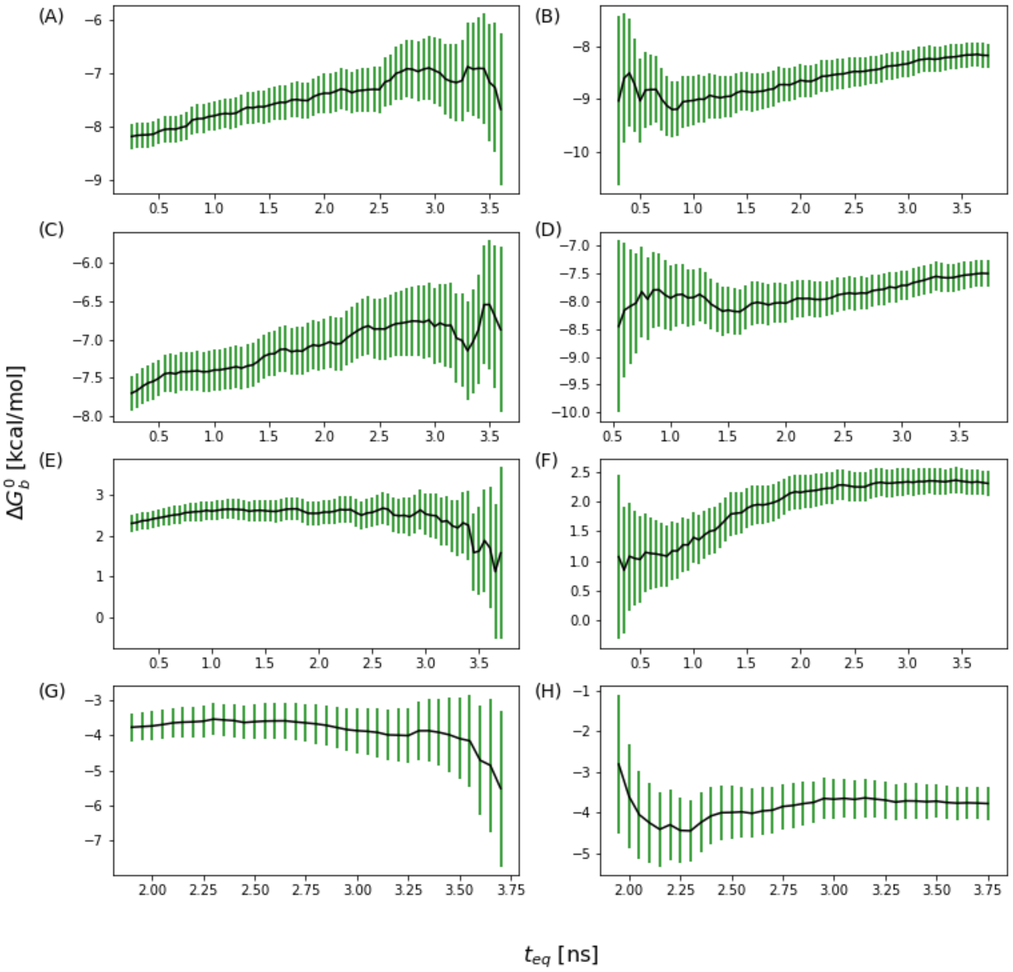

Figure 1: Reverse (A. mutant 1, C. mutant 2, E. mutant 3, G, mutant 4) and Forward (B. mutant 1, D. mutant 2, F. mutant 3, H. mutant 4) cumulative plots for all of the LEDGF peptide mutants after removal of equilibration bias.

Table 1: Table of receptor-peptide interactions broken down by peptide residue. Distances shown are averages across all relevant trajectories. AOP (Atom Of Peptide), AOR (Atom Of Receptor).

Peptide	Residue	% Trajectories	AOP	AOR	RECEPTOR	Distance [Å]
Wildtype	SER 362	93%	100% OG	OE1/OE2	GLU 170	3.12±0.71
	LEU 363	0%				
	LYS 364	100%	NZ	OE1/OE2	GLU 170	2.77±0.46
			19% NZ	O	ALA 169	3.28±0.43
	ILE 365	100%	100% N	O	GLN 168	2.99±0.19
			97% O	O	THR 125	3.37±0.22
			32% O	N	ALA 129	3.52±0.31
	ASP 367	100%	100% OD1	ND1	HIS 171	2.67±0.11
			100% OD1	N	GLU 170	3.24±0.28
			100% OD2	N	GLU 170	2.83±0.15
			100% OD1	OG1	THR 174	2.77±0.29
			27% OD2	O	GLN 168	3.81±0.14
			12% O	O/OG1	THR 125	3.71±0.21
	ASN 367	10%	79% ND2	OE1/OE2	GLU 170	3.42±0.52
	LEU 368	0%				
	ASP 369	0%				
M1:S362A	ALA 362	2%				
	LEU 363	0%	NA	NA		
	LYS 364	100%	NZ	60% OE2, 35% OE1	GLU 170	2.66±0.11
			33% NZ	O	ALA 169	3.23±0.39
			15% O	O	ASP 167	3.44±0.49
	ILE 365	100%	100% N	O	GLN 168	2.96±0.16
			93% O	O	THR 125	3.41±0.26
			46% O	N	ALA 129	3.48±0.27
	ASP 366	100%	87% OD1, 13% OD2	ND1	HIS 171	2.69±0.60
	ASN 367	38%	58% OD1	ND1	HIS 171	3.61±0.26
			30% ND2 30% OE1	OE1	GLN 95	3.38±0.37
			5% N	OG1	THR 125	3.71±0.32
	LEU 368	0%	NA	NA		
	ASP 369	0%	NA	NA		
M2:S362A/L368M	ALA 362	0%	NA	NA		
	LEU 363	0%	NA	NA		
	LYS 364	100%	NZ	50% OE1, 43% OE2	GLU 170	2.72±0.22
			22% NZ	O	ASP 167	3.18±0.48
			13% NZ	O	ALA 169	3.12±0.39
	ILE 365	100%	100% N	O	GLN 168	2.95±0.17
			85% O	O	THR 125	3.44±0.26
			82% O	N	ALA 129	3.41±0.27
			14% O	N	ALA 128	3.81±0.15
	ASP 366	100%	43% OD1	N	GLU 170	3.11±0.25
			85% OD1	ND1	HIS 171	2.73±0.19
			100% OD2	N	GLU 170	2.91±0.18
			20% OD2	OG1	THR 174	2.98±0.55
			16% OD2	ND1	HIS 171	2.75±0.35
			37% O	O	THR 125	3.68±0.26
	ASN 367	56%	70% OD1	ND1	HIS 171	3.69±0.23
			25% ND2	OE1/OE2	GLU 170	??
	MET 368	13%	71% N	OG1	THR 125	3.02±0.24
			29% N	O THR	124	3.52±0.31
	ASP 368	10%	56% OD2	OG1	THR 124	2.82±0.35

Peptide	Residue	% Trajectories	AOP	AOR	RECEPTOR	Distance [Å]
M3:D366N	SER 362	21%	OG	OE1/OE2	GLU 170	3.11±0.70
	LEU 363	0%				
	LYS 364	91%	74% NZ	O	ASP 167	2.92±0.26
			24% NZ	OE1	GLU 170	2.75±0.33
			16% NZ	O	ALA 169	3.64±0.38
			10% NZ	OE2	GLU 170	3.29±0.52
			8% NZ	OG1	THR 125	2.96±0.31
	ILE 365	79%	100% O	O	THR 125	3.36±0.31
			11% O	O	GLN 168	3.44±0.27
			97% N	O	GLN 168	3.16±0.25
			25% O	N	ALA 129	3.60±0.30
	ASN 366	100%	81%		GLU 170	
			37% OD1	N	GLU 170	3.20±0.41
			71% ND2	OE1	GLU 170	3.10±0.45
			53% ND2	OE2	GLU 170	3.14±0.46
			59%		THR 174	
			71% OD1	OG1	THR 174	3.56±0.35
			72% ND2	OG1	THR 174	3.40±0.39
			26% O	O	GLN 95	3.61±0.23
			21% ND2	O	GLN 168	2.75±0.10
			17% ND2	ND1	HIS 171	3.41±0.33
			13% O	OG1	THR 125	3.57±0.28
	ASN 367	57%	82% ND2	OE1/OE2	GLU 170	3.14±0.51
			21%		GLN 95	3.23±0.42
			20% OD1	OG1	THR 174	3.12±0.43
			18%		THR 125	NA
	LEU 368	39%	30% O	OG1	THR 124	3.43±0.35
			30% N	OG1	THR 125	3.15±0.23
			21% N	OE1	GLN 95	3.23±0.27
	ASP 369	18%	61% OD1	N/OG1	THR 174	2.98±0.39
			54% OG1	O	THR 124	3.61±0.22
			50% OD1/OD2	ND1	HIS 171	3.07±0.59
M4:I365A	SER 362	92%	100% OG	OE1/OE2	GLU 170	3.08±0.60
	LEU 363	0%				
	LYS 364	97%	73% NZ	OE1/OE2	GLU 170	2.76±0.47
			34% NZ	O	ASP 167	3.00±0.36
	ALA 365	100%	100% N	O	GLN 168	2.97±0.22
			83% O	N	ALA 129	3.26±0.27
			63% O	O	THR 125	3.40±0.32
			41% O	O	ALA 128	3.64±0.26
	ASP 366	100%	100% OD1/OD2	N/ND1	HIS 171	2.84±0.43
			100% OD1/OD2	N	GLU 170	3.04±0.32
			100% OD1/OD2	OG1	THR 174	2.86±0.60
			55% O	O	THR 125	3.54±0.27
			12% O	O	GLN 95	3.80±0.14
			8% OD1	O	GLN 168	3.78±0.21
	ASN 367	17%	60% ND2	OE1/OE2	GLU 170	3.49±0.6
			53% ND2	O	THR 124	3.35±0.34
			37% ND2	O	THR 125	3.73±0.25
	LEU 368	35%	58% N	OG1	THR 125	3.07±0.26
			35% N	O	THR 124	3.19±0.24
	ASP 369	12%	85% OD1	OG1	THR 124	2.92±0.38
